# Implementing recovery: an analysis of the key technologies in Scotland

**DOI:** 10.1186/1752-4458-5-11

**Published:** 2011-05-15

**Authors:** Jennifer Smith-Merry, Richard Freeman, Steve Sturdy

**Affiliations:** 1 School of Social and Political Science, University of Edinburgh, School of Public Health, University of Sydney; 2 School of Social and Political Science, University of Edinburgh, Room 4.5, 34 Buccleuch Place, EH8 9JS, Edinburgh; 3 ESRC Genomics Policy and Research Forum, University of Edinburgh, St John's Land, Holyrood Road, EH8 8AQ, Edinburgh

## Abstract

**Background:**

Over the past ten years the promotion of recovery has become a stated aim of mental health policies within a number of English speaking countries, including Scotland. Implementation of a recovery approach involves a significant reorientation of mental health services and practices, which often poses significant challenges for reformers. This article examines how four key technologies of recovery have assisted in the move towards the creation of a recovery-oriented mental health system in Scotland.

**Methods:**

Drawing on documentary analysis and a series of interviews we examine the construction and implementation of four key recovery 'technologies' as they have been put to use in Scotland: recovery narratives, the Scottish Recovery Indicator (SRI), Wellness Recovery Action Planning (WRAP) and peer support.

**Results:**

Our findings illuminate how each of these technologies works to instantiate, exemplify and disseminate a 'recovery orientation' at different sites within the mental health system in order to bring about a 'recovery oriented' mental health system. They also enable us to identify some of the factors that facilitate or hinder the effectiveness of those technologies in bringing about a change in how mental health services are delivered in Scotland. These finding provide a basis for some general reflections on the utility of 'recovery technologies' to implement a shift towards recovery in mental health services in Scotland and elsewhere.

**Conclusions:**

Our analysis of this process within the Scottish context will be valuable for policy makers and service coordinators wishing to implement recovery values within their own national mental health systems.

## Background

In recent years, the promotion of recovery has been adopted as a declared aim of mental health policy in a number of countries, including Australia, the United Kingdom and the United States [1-3]. Quite what is meant by recovery in these different settings is difficult to pin down with any degree of specificity. Recovery is not defined by any particular set of therapeutic or preventive practices or services; rather, it represents a more general if rather vague philosophy of mental health care.

However, it is possible to identify a number of key values at the heart of the idea of recovery. Central to these is the idea that recovery is a highly individual process, that is best achieved by utilising an individual's own knowledge and experiences of coping with mental illness and working towards mental wellbeing. Consequently, effective delivery of therapeutic and preventive interventions should be individualised and person-centred, and should privilege the service user's self-knowledge over standardised forms of clinical practice. This in turn is commonly understood to necessitate a radical transformation of mental health systems away from traditional clinical hierarchies, standardised models of care and highly medicalised understandings of mental health and ill-health.

Unsurprisingly, the implementation of these goals has often proved difficult, and critics have argued that in many cases the adoption of recovery in policy discourse has not been matched by a change in mental health practice (e.g. [4]). This has been exacerbated by uncertainty about how recovery values can and should be realised within mental health systems (e.g. [5,6]). Implementing recovery in practice thus remains a significant policy challenge, even in countries that profess a commitment to recovery as a general policy aim.

This paper looks at Scotland as an example of a country where incorporation of recovery into mental health practice has been at least partially successful. As a succession of policy documents shows, Scottish mental health policy has increasingly embraced the concept of recovery over the past five years (e.g.[7-9]). The Scottish Government has expressly set out to implement a recovery oriented mental health system and has invested significant funding and effort in order to achieve this. Drawing on data from interviews and documentary sources, we show that, to the extent that such policy initiatives have been successful in changing the orientation and practices of the mental health services, this has been accomplished through the dissemination of a series of what we call 'recovery technologies'. We use this term to refer to various kinds of techniques, practices and instruments that embody and instantiate the values of recovery, and that provide a means of enacting those values within the mental health system.

Focussing on four such technologies that were identified by our informants as particularly important in promoting recovery in the Scottish context, we examine how those technologies were constructed or imported into Scotland, and how they have been implemented in practice. We also identify some of the factors that our informants see as contributing to or compromising their effectiveness in reorienting Scottish mental health services around the values of recovery.

## Methods

The research presented here is based on data from interviews and primary source documents concerning the development and institutionalisation of recovery in Scotland. The data was collected as part of the large European-wide project, KnowandPol, which has investigated the function of 'knowledge' in relation to health and education policy. Twelve research teams work on this theme at different sites across Europe and the work of our particular team has focused on mental health policy in Scotland. Our interest in the process was therefore with regard to what knowledge was used to produce recovery as a policy, how this knowledge was implemented and what knowledge was created through this implementation process.

Interview respondents were chosen to provide a variety of perspectives on how recovery functions in Scotland. We conducted nine interviews with practitioners (including those working in psychiatry and as service managers), government policy makers, individuals working within non-governmental advocacy organisations and service users/community activists. Based on previous research on the mental health system in Scotland, these roles were selected to provide a good balance of the different kinds of people involved in work on recovery in this context [10]. Interviews were semi-structured and revolved around the history and implementation of recovery from the perspective of the respondent. We analysed the results as we collected the data and stopped interviewing when our data reached saturation in the sense that new interviews were no longer producing new data.

Primary source documents included policy documents, reviews and working papers on recovery, and were created by the Scottish Government, service user groups and non-government organisations working within the Scottish mental health system over the past ten years. All data were entered into the data management software NVivo and then hand-coded according to actor and theme. We ordered the data chronologically and constructed from it a narrative of the development of recovery in Scotland.

From our data we were able to identify four key technologies which have been utilised in the implementation of recovery within Scottish mental health work, and to document how they were developed for use in Scotland and some of the factors that are seen to have enhanced or hindered their implementation in that context.

## Results

### What recovery is, and how it came to Scotland

"Recovery is living. I think it's as simple as that. I think we've complicated it. I just think recovery is getting on with life...I don't think anybody knows [the definition] for a collective. I think every individual knows the answer for themselves." - (Community 1^1^)

We began all of our interviews by asking respondents to define recovery. The establishment of an appropriate definition of recovery has long been a concern for those interested in promoting work on recovery [11]. However, our interviews revealed a striking diversity of views on how recovery might best be defined. Indeed, some interviewees commented explicitly on the multiplicity of definitions and the broadness of the idea, which makes it very difficult to pin down (Community 2; Practitioner 2; Community 1). All agreed, however, that the implementation of recovery in mental health practice is critically dependent on the life situation and history of each individual service user and practitioner. Some spoke of recovery as a process or journey, leading toward a shift in consciousness and practice, while some defined it as a value or set of values that centred around choice and the centrality of the individual (Practitioner 1; Practitioner 2; NGO 1; NGO 2; Community 1; Community 2). This focus on individuals and their personal journeys was in turn seen to imply that the implementation of recovery in the mental health system necessitates a radical restructuring of modes of treatment, hierarchies of knowledge, and the way services relate to service users (Practitioner 1; NGO 1; NGO 2).

Just as our respondents differed in their definitions of recovery, they also offered different stories of where the recovery discourse first originated. However, all emphasised that the concept had been imported into Scotland from elsewhere, either from the US or New Zealand. Our respondents also emphasised that the idea had originally emerged from within the service user movement, and that individual service users and service user groups had been important in creating momentum around the idea in Scotland (NGO 1; Community 2; NGO 2; Community 1; e.g. [12]). Enthusiasm for recovery within these groups eventually led to the idea being incorporated into Scottish mental health policy through the National Programme for Improving Mental Health and Wellbeing. Following the launch of the National Programme in 2003, a 'Scottish Recovery Network' (SRN) was established and funded to progress work and knowledge on recovery. While operating as a quasi-autonomous government agency from the start the SRN has maintained an independence from the Scottish Government through direction from a strong and active board whose membership is dominated by service users and practitioners [13]. From this policy and organisational base, recovery was quickly adopted as a policy priority within a succession of mental health policy documents including *Delivering for Mental Health *(2006), concerned with directing service delivery, and the review of mental health nursing *Rights, Relationships and Recovery *(2006) [14]. Each policy initiative has sought to implement or reaffirm tools and technologies for bringing about a 'recovery oriented' mental health system in Scotland.

### Technologies of recovery

As we have seen, our respondents tended to associate recovery with a set of values that prioritised the individual experiences, needs and choices of service users. Scottish Government policy on recovery deliberately aimed at inculcating these same values into mental health service delivery. As one government actor commented:

"So recovery. Tactically we are using it as a key way to get into the cultures and behaviours of the system...structurally the issues around respect and the quality of the interaction between the people receiving and the people offering the services is going to be the big thing....the first and biggest challenge that we have is to re-humanise services. But recovery, because it is actually dealing with that territory, is a good way into it." - (Government 2)

But this entailed more than simply asserting the ideals and values of recovery in policy documents. It also involved official endorsement and encouragement of a number of 'recovery technologies' that were seen to embody the values associated with recovery, and that provided a means of realising those values within the Scottish mental health system.

Among the practices that our respondents associated with the values of recovery were 'realising recovery training', 'values-based practice' training, a 'narrative project', the Scottish Recovery Indicator, peer support, and Wellness Recovery Action Planning (Practitioner 2; Government 2; Practitioner 1; Community 1; NGO 1; Community 2; NGO 2). Of these, four in particular were seen to have been especially effective in disseminating the values of recovery and in reconfiguring Scottish mental health practice and service delivery around those values. These were recovery narratives, the Scottish Recovery Indicator, Wellness Recovery Action Planning, and peer support. We discuss each of these four technologies separately before moving on to reflect on their utility in orienting the mental health system in Scotland towards recovery.

#### Recovery narratives

The use of narrative work in relation to recovery appears to have originated in New Zealand, in a project undertaken by Hilary Lapsley and others [15]. For this project, researchers supported by the Mental Health Commission collected stories from individual New Zealanders who had experienced from mental ill health and who felt that they had recovered from it to a greater or lesser extent. The resulting report, entitled *"Kia Mauri Tau!" narratives of recovery from disabling mental health problems*, was released in 2002 and was cited as an influential document by several of our respondents (Government 1; Community 2; [16]). The collected narratives served in effect to define and exemplify recovery. Moreover, as the title suggests, they located this specifically in the context of New Zealand culture and identity, in a way that appears to have been highly effective in building a social movement around recovery.

This model was closely followed in Scotland. A narrative research project was initiated by SRN in 2004 and involved collecting narratives of recovery from 64 people across Scotland with lived experience of mental ill-health [17]. The purpose was to "learn" from individual experiences of recovery, share "stories to inspire hope", "offer tools and techniques for recovery", "establish a Scottish evidence base", use this evidence to develop "policy and practice" and "guide and inform the work of SRN" [18]. Such narratives served among other things to demonstrate the effectiveness of recovery; as one of our respondents put it:

"Narratives are an important evidence base." - (NGO 3)

As in the New Zealand case, moreover, our respondents spoke of the importance of SRN's narrative project for developing understanding about recovery within a specifically Scottish context (Government 1; Practitioner 1; Community 2; NGO 3):

"There was a strong focus about...what recovery means in Scotland and the narrative research project run by the Scottish Recovery Network helped to do this." - (Community 2)

Respondents spoke about the narrative project as working to 'Scottishise' recovery by ensuring that the values presented through Scottish recovery work were reflecting what the community of service users understood recovery as (NGO 1; NGO 2; Community 2). This 'Scottish version' of recovery, like the 'New Zealand version' before it, was explicitly presented as distinct and different from an 'American version' of recovery which was portrayed as being "more mono-cultural", less "community centred" and less flexible with regard to personal situations and needs (NGO 1; NGO 2; [19,20]). Interestingly, it is not clear that the approach to recovery that was adopted in America at that time did in fact differ significantly from what was adopted in the Scottish context (Community 2). On the contrary, contemporary American definitions of recovery had much in common with how recovery was represented in New Zealand and Scotland [3] - a situation that one of our respondents attributed to the rapid circulation of a small number of influential texts by authors such as William Anthony [21] and Patricia Deegan [22,23] within a relatively small international community (Community 2). One function of the collection of Scottish recovery narratives thus appears to have been to help to strengthen an indigenous recovery movement by articulating a shared Scottish identity around recovery in contrast to a fictional American 'other'.

The narrative project served as a 'flagship' project which functioned to raise the profile of SRN, and recovery more generally, amongst service users and practitioners (Practitioner 1). It did this by getting individual service users and groups involved in the project, by advertising the project to the mental health service community, and through the wide distribution of the report in different forms. Individual narratives were brought together in a DVD and a book, 20,000 of which had been distributed by mid 2007 (NGO 3). The narratives were also analysed in order to identify and assess the factors which had helped and hindered individual recovery [18]. The findings were released as a report in 2007 and have served to provide a Scottish definition of recovery and an indication of how work on recovery should progress. As SRN has written, "This project has provided the foundation to all of our work" [17].

The narrative approach has also found its way into the practice of 'doing recovery' with service users. One psychiatrist we spoke to mentioned the way that service users were involved in writing "their own story" as part of their journey to recovery (Practitioner 2). Through writing their own stories of recovery, service users identify and reflect on what recovery means in their own lives, thereby internalising the concept, as do practitioners who employ a narrative approach as part of their therapeutic practice. Some services have also developed their own, local narrative projects. For example, the Patient's Council at the Royal Edinburgh Hospital has released its own book, *Stories of Changing Lives*, which has a very similar format to previous narrative publications [24]. As this example shows, narrative work has become an established technology in 'recovery-oriented' mental health services, and works to instantiate and exemplify the concept of recovery in the mental health system in a number of ways. As individual narratives are created by service users and used as a therapeutic tool by practitioners, so the practice and values of recovery are implemented, reproduced and incorporated into the institutional knowledge of the mental health services.

#### The Scottish Recovery Indicator

Another way that the findings of the SRN's narrative project had an impact on mental health service delivery was through the design of the Scottish Recovery Indicator (SRI) (NGO 2). This is a self-assessment tool for measuring the extent to which services are implementing a recovery-oriented practice model in their work. The idea of creating such a tool initially came out of discussions of the implications of the 2006 review of mental health nursing, *Rights, Relationships and Recovery*, and other discussions on "culture and behaviour" that were happening around that time (Government 2; NGO 2). The creation of a recovery indicator for services was seen as necessary in order to clarify what was important and possible in terms of recovery-oriented practice:

"At the time of the nursing review it was thought we were asking a lot of people to do things and people didn't really know what they should be doing and what recovery practice in process was." - (NGO 1)

A proposal to establish a recovery indicator was quickly incorporated into the mental health service policy document *Delivering for Mental Health*, which was released in late 2006. Planning for the tool began immediately and it was piloted in 2007. In 2009 the Scottish Government committed itself to implementing the SRI in *Towards a Mentally Flourishing Scotland: Policy and Action Plan, 2009-2011 *which guides work on population mental health in Scotland [8].

In developing the SRI, careful attention was paid to the specificities of the Scottish context. The SRI tool was modelled on a US tool, the Recovery Oriented Practices Index (ROPI) which had been developed by Anthony Mancini, an academic now working at Columbia University, for use within the New York State Office of Mental Health [25]. ROPI was identified as a suitable starting point for the development of a new indicator for use in Scotland (Government 2). The development of the SRI involved a process of 'Scottishisation' (Community 2; NGO 1) that included not just adopting it to Scottish language and terminology, but also redesigning it to take account of, and make it more amenable to, the kinds of practices associated with recovery in Scotland (NGO 1; NGO 2). Design of the new tool was undertaken under the auspices of the Scottish Recovery Network, who drew on the findings of their own narrative research project to provide an understanding of what recovery meant in a Scottish context (NGO 2). The tool was subsequently tested in several pilot sites to make sure it worked within Scottish settings. As with the narrative project the 'Scottishisation' process that the tool went through contributed to ownership of the concept by those in Scotland.

One particularly striking difference between the SRI and the ROPI was that the SRI was developed for use as a means of self-assessment, rather than for purposes of external evaluation. As one of our respondents observed:

"The ROPI was much more stick than it was carrot...It was an external evaluation and that is the antithesis of recovery for us in Scotland. So it might be that in America you can go and say to a service this is what you are doing wrong and this is what you are doing right but we have a different interpretation of recovery. Recovery is 'you are doing the right thing for you at the right time'." - (NGO 2)

As this quotation demonstrates, self-assessment - as opposed to external audit - was viewed as part of a distinctively Scottish understanding of recovery (Community 2). The SRI thus provides a peculiarly illuminating insight into what recovery has come to mean in relation to Scottish mental health services.

The use of the SRI revolves around online forms which are completed by staff working in services being assessed. Staff are led step by step through the completion of the form, and must answer questions relating to the extent to which recovery practices are evident within different aspects of their services. For example, staff are directed to consider their practices relating to patient assessment in order to ensure they take into account specified aspects of an individual's "basic needs" including housing, nutrition, physical health, entitlements, personal care and spiritual care. Practitioners are directed to look for evidence that appropriate practises are included in their services. The form provides examples of the kinds of evidence that might be considered relevant [26]:

"Entitlements: Service assists with entitlements, advocacy and general advice e.g. legal, financial and housing. (Where indirectly provided, respondents should evidence knowledge of local agencies and offer examples of individual referrals.)

Personal care: Service provides help with personal care, as required. This could include personal hygiene, attention to clothing, haircuts, etc.

Spiritual care: Service records expression of religious or belief-based needs and enables access as required."

Detailed guidelines also make clear just what kind of work is involved in using the SRI. Successful completion of the online forms requires "planning and preparation", including the allocation of tasks between members of the team undertaking the self-assessment. It involves extensive data collection, including review of documents but also interviews with service providers and with current and previous service users, followed by discussion and reflection on the strengths and weaknesses identified and the issues raised. The whole process is expected to take a minimum of thirteen hours to complete. And as the guidelines make clear, this in turn requires "agreement and commitment" from the various staff members taking part [26]. In effect, the whole self-assessment process is designed to immerse the staff completing the SRI within the recovery approach. SRI-based self-assessment is thus a significant step in a mental health service's 'journey' towards a recovery orientation. Several of our respondents argued that, through being obliged to undertake the SRI self-assessment exercise, those working in service provision would reconceptualise their services through a recovery lens, and would thereby come to implement a recovery approach more effectively (Practitioner 2; NGO 2; Practitioner 1). In this respect, the process of completing the SRI was seen to be more important than the data that it produced.

"What we have found in tests and now is that the conversation is everything. So you have a team of people doing it...and having a conversation about it and saying 'well the implications for us are we never talk about strengths.' 'We are an acute admissions ward, why would we want to talk about strengths? They are ill.' 'Well actually when you talk about strengths people talk about what they can do and that creates hope.' 'Oh right. Ok. That's an interesting concept, I hadn't thought of that before'" - (NGO 1)

The use of the SRI technology thus provides a useful way of drawing service providers' attention to aspects of recovery within their own services that they might not otherwise have seen in this light.

At the same time, the data generated by the SRI provide service providers with useful "evidence" about the extent of recovery practices within their organisation. Each organisation can then use this as a baseline against which to improve their performance on the various aspects of recovery identified by the indicator (Practitioner 1). And on a national scale, data produced using the SRI serves to demonstrate that recovery is not merely a vague concept to which lip service is paid, but is actually being implement in Scotland: "the recovery indicator tool has shown us that there is a lot of recovery-oriented work going on but [previously it was] really hard to evidence it because paperwork and the way we document things doesn't reflect that." (Practitioner 1; Practitioner 2).

However, our respondents were at pains to emphasise that the information about services that was generated using the SRI was not 'assessed' by anyone except the services themselves. This self-assessment aspect of the tool was seen to be appropriate because it allowed recovery to grow organically, to be 'owned' by services and integrated thoroughly into practice - a process that could not be rushed (Practitioner 2). Information about services was seen to be most useful if services could use it in their own ways, for improvement of their own work; whereas our respondents feared that resistance to the tool would arise if the data were subject to scrutiny by the government (NGO 1; NGO 2). Indeed, concern had arisen due to government plans to make implementation of the SRI mandatory within services. In response, Government actors were careful to note that they would not monitor the *results *of the SRI, but merely its *use*: while services must undertake self-assessment using the SRI, the results of that self-assessment would be purely for the services' own use (Government 2).

Like the narrative approach, the SRI has been widely adopted as an effective technology for exemplifying and instantiating recovery in Scottish mental health services. However, it has an additional advantage over and above those offered by the narrative approach. By mandating the Scottish Recovery Network to develop a formal self-assessment tool, the Scottish Government were able to instrumentalise the otherwise rather vague values associated with recovery. Government actors saw this 'formalisation' of recovery goals in relation to service provision as an important step in inculcating recovery within Scottish mental health services. In effect, the design of the SRI "took the discussion away from it being a nice cosy service user discussion about 'isn't recovery a nice thing' into something we wanted to directly apply to how we were doing services" (Government 2). By developing the SRI as a mandatory self-assessment tool for service providers, the Scottish Government and the SRN have together produced and disseminated an effective technology for engaging service providers in the process of recovery itself.

#### Wellness Recovery Action Planning

Another 'recovery technology' identified by our respondents as having a significant impact on 'doing' recovery in Scotland was the Wellness Recovery Action Planning (called WRAP for short) (Practitioner 1; Community 1; NGO 1; NGO 2). WRAP originated in America and was devised by Mary Ellen Copeland, herself a service user, whose work on recovery developed out of her own search for personal recovery. It has since been developed as a tool for service users to manage their own recovery by identifying strategies to "restore personal wellness and recovery" [27]. WRAP locates the service user within a larger community of service users, and places a strong emphasis on peer support and the development of an individual recovery plan [28]. By reflecting on how the individual service user has stayed well in the past, and by visualising the strategies that assisted others with their recovery, service users create their own recovery plans that document "daily maintenance, triggers and how to avoid them, warning signs and how to respond to them, and a crisis plan"[29]. According to the Scottish Recovery Network, the use of WRAP in Scotland is underpinned by the "core principles" of "hope", "personal responsibility", "education", "self advocacy" and "support", and individuals "work within these principles to create their own WRAP". SRN adds that that "Each plan should include the following components" [30]:

• Wellness toolbox

• Daily maintenance plan

• Identification of triggers and associated action plan

• Identification of early warning signs and associated action plan

• Identification of signs that things are breaking down and associated action plan

• Crisis planning

• Post crisis planning

As this list demonstrates, the emphasis is on an individual's understanding of their own life. Mirroring the emphasis on self-assessment in the SRI, WRAP is designed as a 'self-management tool'. Also like SRI, WRAP is not assessed, and individuals may "journey" towards recovery at their own pace (Community 1).

As an effective aid to self-management, WRAP was also seen to provide a valuable advertisement for the benefits of the recovery approach, particularly among service users.

"[WRAP] is about how you live your life. All these things are different tools that should be used and the more we use them the more we will see recovery. I think the consumers themselves are the biggest inspiration in it because as they recover other people want a bit of that. Other people want to go on their own journey and recover also." - (Community 1)

However, SRN is careful to stress that "Creating a WRAP can be a challenging process and the process is best supported by a trained facilitator in a group setting" [30]. Consequently, WRAP is chiefly used in the context of inpatient and community mental health services or self-help groups as part of a larger process of planning individual treatment. The WRAP planning process is administered by a facilitator and conducted either on a one-to-one basis or within a group setting [30].

In principle, this process serves to disrupt the traditional practitioner-patient relationship within mental health services by placing the service user's perspective at the centre of treatment/wellness planning. However, our respondents had some concerns that the way WRAP was being rolled out in Scotland might not be best calculated to serve that aim. Specifically, a couple of our respondents felt that too much emphasis was being placed on WRAP as a means of recovery planning, at the expense of other recovery technologies. "WRAP is *one *tool and yet it is being used across the country as if it is *the *tool", said one (Community 1). Another complained that proprietary controls over the use of WRAP had restricted the extent to which it had been rolled out in practice. Copeland, who originally devised WRAP, runs a large non-profit organisation, the Copeland Centre for Wellness and Recovery, and has registered and trademarked the WRAP programme [31]. In consequence, only facilitators who have undertaken official training can offer WRAP training. In Scotland, only one advanced-level trainer is available to train new facilitators (NGO 2; [32]).

Other respondents noted that, while it was originally intended that service users would themselves act as facilitators, in Scotland mental health service staff are also being trained to take up this role (Practitioner 1). One service user commented that this represented "further evidence of the colonisation of a process" that had originally evolved within the service user community; increasingly, this respondent declared, recovery was being made to fit into the structures of mental health services and government policy, and hence was becoming something it was never meant to be (Community 1). Another respondent warned that a problem with current practices relating to WRAP was that it was being instituted "in a traditional way in a traditional environment" (NGO 1). If WRAP was implemented within services that lacked a wider recovery orientation, another respondent feared, it would become "just another care plan" (NGO 2).

These concerns point to an important limit to the effectiveness of WRAP as a recovery technology. If WRAP can only be successfully implemented within a service setting which has already gone through the journey of becoming 'recovery oriented', then it is of little value as a tool for spreading recovery within the Scottish mental health system. At best, it may be used as one among a number of techniques for implementing recovery within services that have already adopted an appropriate orientation. At worst, however, it may be assimilated into more traditional forms of service delivery that fail to embody the aims and values of recovery. Far from assisting in the reorientation of mental health services that recovery is intended to effect, there is thus a risk that WRAP will be appropriated in a process of 'colonisation' whereby the concept of recovery is subverted to the purpose of shoring up the existing social relations of mental health work.

#### Peer support

The final recovery technology discussed at some length by our respondents was the practice of peer support. In the Scottish recovery context, peer support involves the employment within mental health services of individuals with a lived experience of mental ill-health. Such individuals are employed not only to provide experience-based support to service users, but also to exemplify and inculcate the values of recovery within the service itself [33].

The idea of piloting a programme of peer support was first mooted in papers by SRN director Simon Bradstreet in 2005 [34,15]. That year had seen a visit to Scotland by Gene Johnson, a peer support trainer from Arizona. Peer support was also discussed in exchanges related to the International Initiative for Mental Health Leadership (IIMHL) conference in New Zealand in 2005 (Community 2). This was followed in 2006 by a literature review and a national conference on peer support which aimed to answer the questions: 'what can we learn about peer support to enable Scotland to do it itself?' and 'how can we get people to understand peer support enough to do it?' (NGO 1). The idea was adopted into policy in 2007, when the document *Delivering for Mental Health *committed the Scottish Government to piloting the paid employment of peer support workers [7].

The Support Worker Pilot programme was finally undertaken in 2008-2009 by the Scottish Government and SRN, and was implemented in five health boards. Most of the peer support workers had no defined role when they were first employed; rather, they worked with services to determine what an appropriate role might be. Across the pilot sites, two main facets of the role were identified: "sharing lived experience" and "modelling recovery" [35]. Bradstreet and Pratt have since described the peer support role as "offering mutuality, empowerment, modelling hope and the sharing of lived experience with service users" [33].

In fulfilling this role, peer support workers impact on services by demonstrating that recovery is possible. From a service users' perspective, peer support workers do indeed seem to provide effective models of recovery, and thereby give hope that recovery is possible [35]. At the same time, the employment of current or past service users within a service setting also works to endorse the individual, experience-based knowledge that service users themselves possess. As such, it serves to validate other key tools of recovery such as WRAP, which emphasise the importance of self-knowledge and self-management. One respondent noted the radical way that this has impacted on services:

"They now employ acute inpatient forum workers who are peer support workers and that has shifted things around a little. A user organisation is now placing peer support workers in acute units so they can assess patient experience and it has very much come from a local recovery network." - (NGO 1)

In effect, peer support workers help to challenge traditional hierarchies within mental health services by acting as a disruptive force to change the culture of mental health service delivery (NGO 1). Moreover, the Peer Support Pilot project indicated that the presence of peer support workers were effective in helping to reorient services toward recovery "values" (Practitioner 1; [33,35]). Reflecting on their experience of the pilot project within their own health board area, one respondent commented: "the peer support pilot...was hugely beneficial in the areas that the peer support workers were working in looking at things like the language and the ethos of recovery" (Practitioner 1). The employment of peer support workers was also seen as beneficial in working to break down the "us and them" mentality whereby staff assumed that they held all the power and knowledge in the system, while service users were treated as the objects of staff knowledge [33].

However, the evaluation of the pilot program also noted that while the peer support workers had helped to move the service toward a recovery orientation, their work had been made much more difficult where those services were not already moving in that direction. This placed a heavy burden on service users to pull the system into shape - and expectation that the evaluation judged to be problematic [33,35]. As with the use of WRAP, the effectiveness of peer support to effect a change in the orientation of 'traditional' mental health services was seen to be limited in the absence of other measures to implement a recovery approach.

## Discussion

From the Scottish point of view, recovery is a doubly imported concept: imported into Scotland from different international contexts; and imported from the service user community into the world of policy and practice. Yet that importation has enjoyed a significant degree of success. This has been achieved through the creation of a number of recovery technologies which have helped to disseminate recovery within its new environment. Each of the four technologies that we have focussed on here works towards the same ends of instantiating recovery as a practice within the Scottish mental health system, and of exemplifying the meaning and values of recovery to service users and practitioners elsewhere within that system.

The efficacy of recovery narratives as a recovery technology lies especially in their ability to exemplify and publicise what recovery means to service users, and in providing accessible and easily disseminated evidence that recovery works. Narratives drawn from the lives of local people also help to give a distinctly local flavour to the idea of recovery, and encourages 'ownership' of the recovery approach by the population at which it is aimed. As we have seen, the stories collected by the Scottish Recovery Network's narrative project have been used to construct an account of a distinctly Scottish version of recovery. Whether or not this version really differs from how recovery is enacted in other countries is beside the point. What matters is that, through the practices of telling and recording Scottish stories of recovery, the actors involved have come to regard recovery as a national achievement, which expresses the distinct character of the Scottish people and their ability to cope with mental illness. The wide distribution of these narratives among both service user and practitioner networks enables other Scots to appreciate the relevance of recovery to their own lives. That appreciation is reinforced, moreover, by the incorporation of narrative methods into therapy, whereby service users are encouraged to narrate their own stories of recovery, thereby expanding the community of individuals for whom the idea of recovery provides a means of making sense of their own lived experience of mental illness and mental health.

If recovery narratives act chiefly by giving a voice to service users, the Scottish Recovery Indicator specifically targets service providers. By specifying a highly disciplined process of self-assessment and collective reflection, the SRI serves to make apparent some of the key principles, practices and values associated with the promotion of recovery within mental health service provision. Through the activities and 'conversations' that must be undertaken when completing the requisite forms, the concept of recovery is firmly imprinted in the minds of those using the SRI. As a means of disseminating a recovery perspective within the mental health services, moreover, the SRI has the additional advantage of being endorsed both by government and by the Scottish Recovery Network. At the same time, it retains an element of voluntarism and freedom from external evaluation - values that, like the narrative research that informed the design of the SRI, are seen to be distinctively Scottish in contrast to American recovery assessment tools.

The fact that the SRI is seen to have been adapted in accordance with specifically Scottish ideals of recovery also contrasts with the career of another imported recovery technology, namely Wellness Recovery Action Planning. Like the use of recovery narratives as a therapeutic technique, WRAP is intended as a means of disseminating recovery amongst both service users and practitioners by reconfiguring the relationships which mediate care and treatment. Using WRAP in service delivery, the service user's experience, self-knowledge and powers of self-determination are made central to therapeutic interactions, with implications for how both the service user and the practitioner envisage their own roles. However, the fact that WRAP is a trademarked technology means that Scottish recovery advocates have not been able to adapt it to their own ideals as they did with the SRI. Meanwhile, some recovery advocates voice fears that WRAP is being re-appropriated into more traditional, practitioner-dominated models of care that run counter to the values of recovery. However well-intentioned WRAP may be as a recovery technology, its efficacy appears to have been compromised, at least in the Scottish situation, by legal restrictions on the extent to which it can be owned by service users - an ironic outcome in view of WRAP's professed intention of empowering users.

No such problems attach to the appointment of service users as peer support workers on the staff of the mental health services as a means of orienting services towards recovery values. As with WRAP, the placement of peer support workers within services is intended to disrupt entrenched expectations about staff-patient relationships positions and the validity of service user knowledge in the context of treatment. The fact that peer support workers function well in this role also serves to demonstrate the validity of the recovery concept: in effect, peer support workers act as living embodiments of recovery. The only doubts that our respondents voiced about peer support related to how much can be achieved by such means, in the absence of more systematic efforts to reorient service delivery. Ultimately, the implementation of recovery requires holistic change in the organisation and social relations of mental health services and in the understanding of mental health and illness that informs those services. Placing individual service users as peer support workers within mental health services may serve to exemplify some of the values of recovery, but such workers will rarely be in a position to catalyse the kind of wholesale restructuring of practice and understanding that will be necessary to fully implement recovery.

Our argument in this paper has been that the successful enactment of recovery's key technologies in Scotland has meant that the 'recovery discourse' has become a hegemonic one guiding the operation of the mental health system. Indeed, not one of our respondents could point to an area within the system that was fully resistant to recovery. Even within psychiatry, where it was supposed some areas of resistance might lie, respondents felt that any resistance resulted from a lack of understanding about recovery and that any mis-understanding psychiatrists might have about recovery was slowly changing as the recovery discourse came to pervade the system and its operation [Practitioner 2; NGO 2; Community 2; NGO 1; Community 1]. It is interesting to note, then, that this success has provided, for our respondents, one of the major sources of worry about recovery. The recovery discourse has become so central in Scotland that it is in fact "a difficult thing to argue against" [NGO 2]. One respondent commented:

"There has been a criticism of the work.... It is impossible to disagree with so as soon as you do argue with it you are seen as some sort of evil anti-helping people type person when you are actually trying to make a cogent point. It's almost too PC to disagree with and I don't think that is helpful." - (NGO 1)

This is a considerable problem because, as this respondent noted, it means that valid critiques of recovery are not heard. For this reason, some of those individuals who were most integral to the initial move of recovery from social movement into policy are starting to think "beyond recovery" and push for new perspectives to enter the mental health discourse [Community 1]. A recent article by Cowan and Tilley [14] offers a more in-depth critique of recovery along this vein and will provide a good basis for further work in the area.

## Conclusions

This paper has examined the way that four specific 'recovery technologies' have been devised and implemented with the aim of disseminating recovery through the mental health system in Scotland. The Scottish case has been presented not so much as an exemplar of recovery practice, but as an example of a system which has sought to implement recovery in an holistic way and the technologies which have been put in place to do this. We have shown how each recovery technology acts in different ways to instantiate and exemplify the meaning of recovery at different sites within the mental health system. And we have looked at some of the factors influencing the effectiveness of those different technologies in promoting change within that system as a whole. We are now in a position to venture some more general conclusions about the role of recovery technologies in fulfilling the aim of implementing recovery within the Scottish mental health system.

One implication of thinking about the different tools and initiatives discussed in this paper as 'technologies' for the dissemination of recovery is to draw attention to the ability of those technologies to move from one location within the mental health system to another. In order to effectively instantiate and exemplify recovery at different sites in the system, those technologies must remain relatively stable as they reproduced or re-enacted at different locations. All the technologies that we have discussed have something of this character. Recovery narratives collected through the Scottish Recovery Network's narrative research project are distributed as printed pamphlets or through online resources, while the collection of new narratives from particular locales has been modelled on the SRN's methodology. The Scottish Recovery Indicator provides a standardised tool that can be accessed and implemented in similar ways within different service settings. The same can be said of WRAP. The employment of peer support workers, meanwhile, provides a way in which individuals who personally embody the values of recovery can be appointed to work in diverse locations within the mental health services.

At the same time, it is clear that these different technologies serve to instantiate recovery in rather different ways. This is important, since the effective implementation of recovery implies holistic change both in the provision of mental health services and in the expectations of service providers and service users alike. The ability of any one technology to achieve such holistic change within an established and entrenched system of mental health care is likely to be limited, as indeed our respondents noted with respect to the impact of peer support workers. Rather, such change is much more likely to be effected through the influence of multiple recovery technologies acting on different parts of the system. In this respect, the vagueness with which the overarching concept of recovery is itself defined, and the tendency to talk about it as a rather loose set of values rather than as a particular body of practices, actually appears as something of an advantage. Recovery, it seems, can be interpreted and exemplified through a diversity of practices and processes. In effect, this loose definition of recovery permits the development of multiple tools and technologies that can be used to import the values of recovery into the mental health system in multiple ways and at multiple sites. A similar point holds true if we think of the geographical importation of recovery into Scotland. Here too, the interpretative flexibility surrounding the meaning of recovery appears to have been beneficial, with the collection of indigenous Scottish recovery narratives and the adaptation of the Scottish Recovery Indicator to the needs of local services both contributing to the sense of a distinctly Scottish version of recovery that has helped to promote 'ownership' of the concept amongst both service users and service providers.

In effect, the importation of recovery into the Scottish mental health services has involved striking a balance between exploiting the diversity of interpretative possibilities permitted by the rather vague definition of recovery itself, and maintaining the stability of the different technologies that have been adopted as a means of instantiating and exemplifying recovery in practice. The maintenance of such a balance is not inherent in the concept of recovery itself; rather, it must be achieved through the assertion of a degree of social control over the ways that recovery is interpreted and implemented. In this respect, the role of the Scottish Recovery Network appears to have been crucial. As an organisation that brings together a community of service users and service providers with an interest in promoting recovery in Scotland, SRN itself embodies many of the key values of recovery. Moreover, with official backing from the Scottish Government, SRN has been instrumental in designing, domesticating and disseminating three of the four recovery technologies that we have considered in this paper, namely recovery narratives, the Scottish Recovery Indicator and peer support. Indeed, it is striking that the one recovery technology about which our respondents voiced serious misgivings, namely WRAP, is the only which over which, for proprietary reasons, SRN has been unable to exert significant control.

The key place of individual experience within definitions of 'recovery' and the technologies that we have described raises some issues about the possibilities for the effective implementation of recovery. One of the government respondents, quoted above, spoke of "using" the 'recovery' discourse to get at the "cultures and behaviours of the system". This aim, along with the stated problems faced by peer support workers in their task of performing or modelling recovery within services, draws attention to the issues involved in implementing an individual-focused concept such as recovery to address entrenched systemic issues. While Scotland professes an 'inequalities' approach to all policy, mental health included, the 'recovery' programs itself has not yet addressed this area in any substantive ways [36]. Recent work by several scholars has drawn attention to this issue [37,38]. Hopper [38], drawing on the work of Amartya Sen, has addressed this through describing the possibilities raised by linking 'recovery' with a 'capabilities' approach which addresses the *individual *social and structural context of need. This approach, although complex and difficult to operationalise [37], might be a useful starting point for thinking about the ways technologies for a 'recovery-oriented' mental health system might also address the systematic inequalities that entrench disadvantage and bring about 'social' as well as individual 'recovery'. Further research is needed which teases out this complex issue.

Our findings have implications for the kinds of strategies that might best be adopted by policy makers and activists working to implement a recovery-oriented mental health system, whether in Scotland or elsewhere. As we have seen, recovery is generally defined in terms of the kinds of values that should pervade the provision of mental health care, and the way that service providers and service users conceive of their respective roles and relationships. But that definition says little about how such values and changes in role can best be achieved in practice. Our findings indicated that the holistic reorientation of service provision around the aims of recovery will likely depend upon the promotion and dissemination of multiple recovery technologies tailored to the local peculiarities of mental health care, and acting in different ways and at different sites in the system. Without such technologies, the meaning of recovery as a set of values will remain unrealised in practice. But if the overall effect of those technologies is indeed to be a holistic change in service provision, some measure of control will need to be asserted over the way that they are interpreted and implemented in practice. In the absence of such control, there is a serious risk that the piecemeal implementation of recovery technologies will simply result in their appropriation into more traditional structures and social relations of mental health care. In the Scottish setting, this control has been exerted by the Scottish Recovery Network, with backing from the Scottish Government. Whether similar models might best be adopted elsewhere, or whether other approaches will prove more appropriate to other local and national settings, is a matter for conjecture and experimentation.

## Competing interests

The authors declare that they have no competing interests.

## Authors' contributions

JSM collected and analysed the data and constructed the first draft of the paper. RF and SS made substantive contributions to the structure and arguments made within the paper. All authors approved the final draft.

## List of abbreviations

Throughout this paper we refer to respondents using a signifier that combines the category which best represents the position they were speaking from, plus a numerical identifier: e.g. 'NGO 2'. Although we are aware that individuals occupy multiple positions in the mental health community, for example as both government worker and community activist or NGO worker and service user, we have made these distinctions for clarity of referencing. ROPI: Recovery Oriented Practice Indicator; SRI: Scottish Recovery Indicator; SRN: Scottish Recovery Network; WRAP: Wellness Recovery Action Planning.
